# Knowledge, Perception, and Clinical Experiences on Molar Incisor Hypomineralization Amongst Dental Professionals: A Systematic Review and Meta-Analysis

**DOI:** 10.3390/jcm15145591

**Published:** 2026-07-16

**Authors:** Gabriela Balixa, Carlota Rodrigues, João Botelho, Vanessa Machado, Luísa Bandeira Lopes

**Affiliations:** Egas Moniz Center for Interdisciplinary Research (CiiEM), Egas Moniz School of Health & Science, Caparica, 2829-511 Almada, Portugalcarlotarodriigues05@gmail.com (C.R.);

**Keywords:** Molar–incisor hypomineralization, knowledge, perception, attitude, awareness, dental professionals

## Abstract

**Background:** Molar–incisor hypomineralization (MIH) is a common developmental enamel defect that presents important diagnostic and therapeutic challenges in pediatric dentistry. Differences in dental professionals’ knowledge and clinical confidence may affect patient care. **Aim:** To evaluate dental professionals’ awareness, diagnostic confidence, clinical management, referral practices, and training related to MIH. **Methods:** A systematic review and meta-analysis were conducted in accordance with PRISMA guidelines. Random-effects meta-analyses were performed to calculate pooled estimates, with subgroup analyses by geographic region, dental specialty, and risk of bias. **Results:** Thirty-six observational studies including over 10,000 dental professionals were included. Awareness of MIH diagnostic criteria was moderate (72.1%) and higher among pediatric dentists than general dental practitioners. Diagnostic confidence (67.5%) and comfort in providing treatment (64.1%) were suboptimal, particularly among non-specialists. Most respondents perceived a distinct caries pattern in MIH-affected teeth (84.8%), while referral to pediatric dentists was inconsistent (52.5%). Approximately 80% of participants reported a need for additional MIH-related training. Substantial heterogeneity was observed across analyses. **Conclusions:** Despite moderate awareness of MIH, important gaps persist in diagnostic confidence, clinical management, and referral practices. Strengthening undergraduate education, continuing professional development, and structured referral pathways is essential to improve early diagnosis, appropriate management, and outcomes for children affected by MIH, highlighting the pivotal role of paediatric dentists in interdisciplinary care.

## 1. Introduction

Molar–incisor hypomineralization (MIH) is a qualitative developmental enamel defect. The etiology and mechanism of its development are still unknown, although multiple systemic, genetic, epigenetic, and environmental factors have been associated with MIH, suggesting a multifactorial etiological model. Clinically, it manifests as well-demarcated opacities ranging in color from white to light brown, depending on the degree of hypomineralization. By definition, MIH affects one or more permanent molars and, in some cases, incisors [1]. However, similar demarcated opacities may also appear on other teeth, reflecting a broader spectrum of enamel hypomineralization.

Managing MIH poses significant challenges for both clinicians and the affected children. Depending on its severity, MIH is frequently associated with enamel fragility, post-eruptive breakdown, increased susceptibility to caries, restoration failure, and marked hypersensitivity, which often causes considerable discomfort [2]. During dental care, these clinical manifestations are commonly accompanied by behavioral management difficulties and elevated dental fear or anxiety [3].

Moreover, MIH-affected teeth often exhibit reduced anesthetic efficacy and compromised bonding performance of restorative materials [4,5,6,7]. These limitations contribute to higher rates of treatment failure and the need for repeated restorative interventions [8,9]. Therefore, therapeutic approaches vary widely, from preventive and desensitizing strategies to extensive restorative treatments or, in severe cases, extractions followed by orthodontic rehabilitation.

Early diagnosis and preventive management initiated at the time of eruption are key to mitigating complications and preserving the tooth structure. However, evidence indicates that knowledge and confidence regarding MIH remain insufficient among dental professionals worldwide [10]. Accurate diagnosis requires familiarity with developmental defects of enamel (DDE) and the ability to differentiate MIH from other enamel anomalies, an area that continues to present uncertainty in clinical practice.

Given the global prevalence of MIH, estimated at approximately 13% [11,12], and its substantial clinical implications, it is essential that dental practitioners possess the knowledge and skills to ensure accurate diagnosis and effective and standardized management. Our aim is to provide a comprehensive global assessment of dental professionals’ knowledge, diagnostic confidence, clinical experience, and perceptions related to MIH, allowing comparisons through meta-analysis quantitative estimates.

## 2. Materials and Methods

### 2.1. Protocol and Registration

This systematic review protocol was designed and approved by all authors and registered in the National Institute for Health Research PROSPERO, International Prospective Register of Systematic Review (http://www.crd.york.ac.uk/PROSPERO, in 6 September 2025; registration ID Number: CRD420251081430). The completed PRISMA 2020 checklist is provided in the Supplementary Materials as Table S15.

The review was conducted following the Preferred Reporting Items for Systematic Reviews and Meta-Analyses (PRISMA) guidelines [13].

### 2.2. Focused Question and Eligibility Criteria

This review addressed the following PECO question:

“What is the knowledge, perception, and clinical experience regarding MIH among dental professionals?”

The corresponding criteria were defined as follows:

- Population (P): Dental professionals.
- Exposure (E): Self-reported knowledge, awareness, perceptions, confidence, and clinical experience related to MIH.
- Comparison (C): No predefined comparison group was established; however, subgroup differences (e.g., between postgraduate students and general practice dentists or among different specialties) were extracted when available.
- Outcome (O): Level of knowledge, diagnostic accuracy, perceived confidence, and clinical management practices regarding MIH.

Studies were eligible for inclusion if they met the following conditions: (1) Observational studies (cross-sectional) employing structured questionnaires assessing knowledge, perception, or clinical experience related to MIH; (2) studies involving dental professionals (dentists, specialists, hygienists, therapists, mixed professional groups) and postgraduate dental students. The exclusion criteria comprised studies that did not include dental professionals or focused only on undergraduate dental students; publications without fully accessible data; manuscripts lacking a complete peer review; and studies that evaluated enamel defects without specific reference to MIH. Reviews, case reports, editorials, commentaries, qualitative studies, and conference abstracts were also excluded.

### 2.3. Search Strategy

Relevant studies were identified using a detailed search strategy developed for each database (PubMed, Web of Science, Embase and LILACS, from inception until April 2025). The search algorithm was based on the following terms:

(“Molar Incisor Hypomineralization” OR “Molar Hypomineralization” OR “MIH” OR “hypomineralised molars” OR “hypomineralized molars” OR “Developmental defects of enamel” OR “Enamel defects” OR “Amelogenesis imperfecta” OR “Dental fluorosis”) AND (“Knowledge” OR “Awareness” OR “Perception” OR “Attitude” OR “Practice”).

Language restrictions were applied, with inclusion limited to publications written in English, while no restrictions were imposed on the publication year. Additionally, Grey literature was searched via http://www.opengrey.eu/; accessed on 1 January 2020.

The search results were uploaded into Rayyan, (web-based platform; accessed 25 April 2025) an AI-powered systematic review management platform [14], and a second check for duplicates was performed. DedupEndNote (Version 1.0.0) was used for automated deduplication [15].

### 2.4. Study Selection

Two independent reviewers (GB and LBL) screened the titles and abstracts. Full-text evaluation was performed for all studies judged to be potentially eligible by at least one reviewer. Disagreements were resolved through discussion or consultation with a third reviewer (JB). Inter-rater reliability was assessed using Cohen’s kappa statistics, which were calculated separately for title/abstract screening and full-text assessment.

### 2.5. Data Extraction Process and Data Items

Data extraction was conducted independently and in duplicate by two reviewers (GB and LBL). The level of agreement between the reviewers was evaluated using kappa statistics. Disagreements were resolved through discussions with a third reviewer (JB).

The following key information was extracted from each eligible study:

- General study details: author, year, type and validation status of the questionnaire.
- Sampling characteristics: Country, sample size, and dental professional category (Pediatric Dentists, General Dental Practitioners, other dental specialties, and Dental hygienists/therapists).
- Study outcomes: self-reported knowledge or awareness of MIH diagnostic criteria, perceived differences in caries patterns, diagnostic confidence, comfort in providing treatment, referral practices, and perceived need for further MIH-related training.
- Methodological aspects: Research design and measurement tools and validation of questionnaires.
- Funding sources: Information on financial support, if available, should be provided.

### 2.6. Risk of Bias (RoB) Assessment

The Risk of bias in the included cross-sectional studies was evaluated using an adapted version of the Newcastle–Ottawa Scale (NOS) for observational studies. Calibration between reviewers was performed using a pilot test of 10 randomly selected studies, and reliability was measured using Cohen’s kappa. Any doubts or discrepancies were resolved through discussion and consensus with a third author (JB). This tool includes three domains: selection, comparability, and outcome assessment and categorizes studies as follows: Low RoB (7–9 stars); Moderate RoB: (5–6 stars); and High RoB (<5 stars) [16,17,18].

### 2.7. Summary Measures and Synthesis of Results

A set of a priori sensitivity analyses was performed to determine whether studies with a low risk of bias reported different values compared to studies with a moderate-to-high risk of bias. If such a difference was observed, we reported separate estimates; otherwise, we reported an overall estimate.

Predefined tables also registered the number of participants, mean, and standard deviation (SD) values. Random-effects meta-analysis and forest plots of prevalence were calculated in R version 4.1.0 (R Studio Team 2018) using ‘meta’ package [18], through DerSimonian–Laird random-effects meta-analysis.

Sensitivity analysis was planned to explore whether the risk of bias could influence the overall pooled estimates. In the case of a significant result, we planned to conduct pooled estimates according to the level of RoB achieved. Heterogeneity among the studies was assessed using I2 test statistics (*p*  <  0.1) and Egger’s significance test, respectively [11]. A high level of heterogeneity was identified when the I2 statistic surpassed 50% [11]. In meta-analyses that included 10 or more studies, publication bias was evaluated [11]. Publication bias was assessed in pooled estimates comprising 10 or more studies using Egger’s test [19]. All tests were two-tailed, with an alpha set at 0.05. Estimates were described using a 95% confidence interval (CI).

## 3. Results

### 3.1. Study Selection

**Figure 1:**
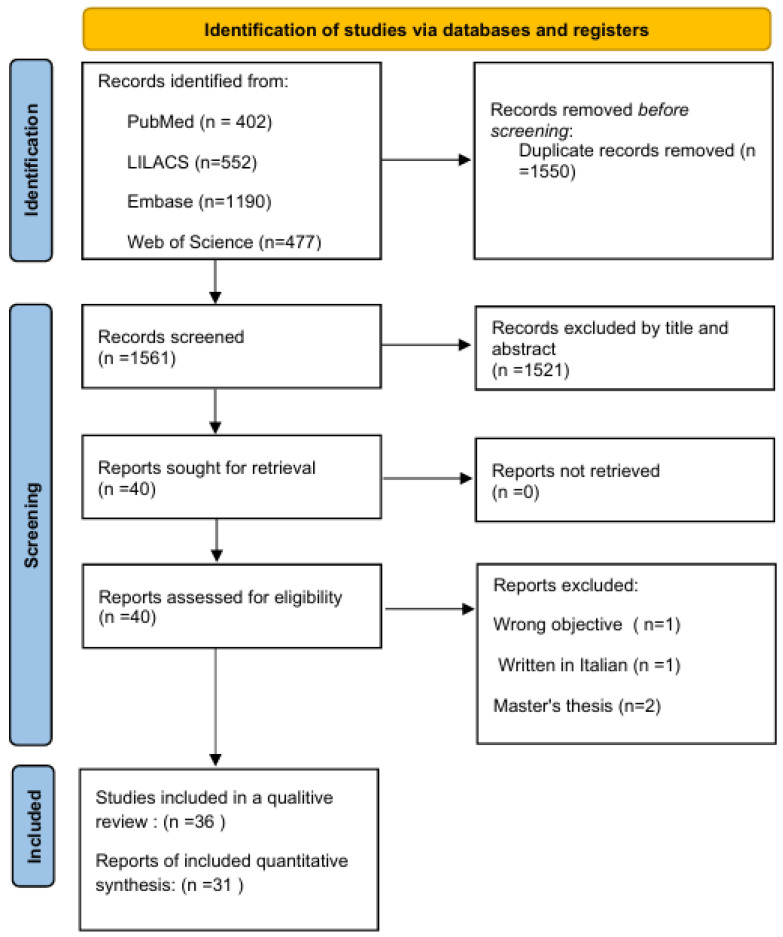
PRISMA Flowchart.

The online search identified 2621 publications. After removing duplicates, 1521 studies were excluded based on their titles or abstracts. Forty articles were eligible for full-text review, of which four were excluded (Supplementary Table S1). A total of 36 observational studies were included in the qualitative synthesis. Inter-observer reliability for full-text screening was excellent (kappa = 0.83, 95% CI 0.79–0.87) (Figure 1).

### 3.2. Studies Characteristics

In total, 15,113 oral health professionals were included in the study. The participants were general dental practitioners (3921), pediatric dentists (1503), and other dental specialists (948). Most studies (26) allowed for a comparison between different professional profiles.

The studies were conducted across 29 countries, across the following regions: Europe, Oceania, the Americas, Asia, and the Middle East. All studies were published between 2008 and 2024 and employed a cross-sectional design (Supplementary Table S2).

The main outcomes assessed were knowledge of the diagnostic criteria and the perception of different patterns of caries in MIH teeth, confidence in the ability to diagnose and provide treatment in these cases, likelihood of referral to a pediatric dentist, and demand for additional information and training on MIH. Data were primarily collected using self-reported questionnaires (Table 1).

### 3.3. Methodological Quality

Overall, the studies presented a low to moderate risk of bias (Supplementary Tables S3–S8). Most studies scored well in the Selection domain, demonstrating clearly defined study populations, appropriate sampling strategies, and adequate sample sizes. The Comparability domain showed greater variability, as only a subset of studies adjusted analyses for relevant confounders, such as dental specialty or professional experience. In the Outcome domain, most studies used structured questionnaires and appropriate statistical analyses; however, outcomes were predominantly self-reported, which may have introduced reporting bias.

### 3.4. Synthesis of Results

We first confirmed whether the RoB influenced the overall pooled estimates. None of the pooled subgroup meta-analyses showed that RoB significantly influenced the computed estimates (Supplementary Tables S3–S8). Thus, we pooled estimates combining both studies with low and moderate-to-high risk of bias.

### 3.5. Knowledge and Awareness for MIH Diagnostic Criteria

Overall, 20 studies provided data for synthesis, with 10,181 dentists involved (Supplementary Tables S3–S8). Sensitivity analysis showed that the risk of bias did not substantially influence the overall outcome (Q = 2.15; df = 1; *p* = 0.1423) (Table 2). The overall pooled estimate of awareness of MIH diagnostic criteria was 72.1% (95% CI: 59.3–82.1; *p* < 0.0001; I^2^ = 99.0%). However, the considerable between-study heterogeneity (I^2^ = 99.0%) suggests that this estimate should be interpreted with caution. When stratified by region, higher knowledge was reported in studies from Oceania (90.2%) and America (84.3%) than in Europe (69.4%) and Asia (66.0%). Diagnostic assurance was highest among PDs (94.8%; 95% CI: 84.4–98.4), followed by ODS (74.4%; 95% CI: 56.4–86.7) and GDPs (67.3%; 95% CI: 50.7–80.5), with significant heterogeneity across the studies (I^2^ = 87.6–98.4%).

### 3.6. Perception of Caries Pattern in MIH Compared to Non-MIH

Overall, 12 studies (totaling 2536 dentists) reported data on the perceived caries pattern in MIH compared to non-MIH counterparts. Overall, 64.1% (95% CI: 55.3–72.1; *p* < 0.0001; I^2^ = 95.1%) reported that people living with MIH present a different pattern of caries than their healthy counterparts. This perception ranged between 58.2–83.0 according to the geographic region (Supplementary Table S10), and PDs reported the highest perception, of 88.4% (95% CI: 84.1–91.6; I^2^ = 34.6), 20% or more than GDPs (63.4%, 95% CI: 53.2–72.6; I^2^ = 93.6) or ODS (53.7%, 95% CI: 31.1–74.9; I^2^ = 96.1).

### 3.7. Comfortable Providing Treatment to Children with MIH

Overall, 15 studies (totaling 2536 dentists) reported data on comfort in providing treatment to children with MIH. Overall, 67.5% (95% CI: 58.2–75.6; *p* < 0.0001; I^2^ = 97.5%) of the participants reported feeling comfortable treating children with MIH. This perception varied by region, ranging from 54.4% in Asia to 92.9% in Oceania, with intermediate values in America (76.5%) and Europe (74.3%) (Supplementary Table S11).

Regarding professional specialty, PDs reported the highest comfort levels (92.1%, 95% CI: 84.3–96.2; I^2^ = 83.0), nearly 30% higher than GDPs (63.3%, 95% CI: 52.1–73.2; I^2^ = 96.6) and ODS (64.0%, 95% CI: 45.0–79.4; I^2^ = 95.7).

### 3.8. Refer Children with MIH to a Pediatric Dentist

Overall, 10 studies (totaling 2907 dentists) reported data on referral patterns in children with MIH. The pooled estimate indicated that 52.5% (95% CI: 34.7–69.6; *p* < 0.0001; I^2^ = 97.5%) of the participants reported referring children with MIH to a pediatric dentist. This perception ranged from 42.0% in Europe to 83.0% in Oceania, with America (73.2%) and Asia (57.2%) showing intermediate proportions (Supplementary Table S12).

When stratified by specialty, ODS reported the highest referral proportion (84.1%, 95% CI: 68.4–92.8; I^2^ = 90.7), approximately 25% higher than GDPs (58.5%, 95% CI: 45.8–70.1; I^2^ = 95.2). About 59.7% of PDs (59.7%, 95% CI: 28.1–84.9; I^2^ = 97.5) reported referring to other PDs. However, the primary studies did not report the clinical circumstances underlying these referrals. Therefore, although this finding indicates that referrals do occur, the reasons underlying this practice could not be determined from the available evidence.

### 3.9. Confidence in the Diagnosis of MIH

Collectively, 13 studies (5784 dentists) reported data on confidence in diagnosing MIH. Overall, 67.5% (95% CI: 58.2–75.6; *p* < 0.0001; I^2^ = 97.5%) of the respondents reported being confident/very confident in diagnosing MIH. This confidence varied across regions, with higher levels in Oceania (92.9%), America (76.5%), and Europe (74.3%) and lower levels in Asia (54.4%) (Supplementary Table S13).

According to professional specialty, PDs reported the highest confidence (92.1%, 95% CI: 84.3–96.2; I^2^ = 83.0), while ODS (64.0%, 95% CI: 45.0–79.4; I^2^ = 95.7) and GDPs (63.3%, 95% CI: 52.1–73.2; I^2^ = 96.6) reported substantially lower levels.

### 3.10. Training

In summary, 14 studies (4522 participants) reported data on MIH training. The pooled estimate showed that 80.7% of participants reported a need for further MIH-related training (95% CI: 72.3–87.2; I^2^ = 95.0). This perception varied geographically, being highest in Asia (83.2%) and Europe (74.4%) and lower in America (58.2%) (Supplementary Table S14).

Regarding professional specialty, GDPs reported the highest proportion (90.5%, 95% CI: 76.0–96.6; I^2^ = 95.4), followed by PDs (83.0%, 95% CI: 66.3–92.4; I^2^ = 91.0). ODS had the lowest estimate (64.7%, 95% CI: 42.0–82.2; I^2^ = 96.3), with substantial heterogeneity observed across all subgroups.

### 3.11. Other Analyses

Visual assessment of funnel plots with Egger’s regression tests revealed variable evidence of publication bias across the outcomes. Significant small-study effects were detected for awareness of MIH (Figures S1–S3), pattern of caries in MIH compared to non-MIH (Figures S4 and S5), and confidence in diagnosing MIH in the overall data and regarding PDs (Figures S10–S13, suggesting possible publication bias in these domains. In contrast, no significant publication bias was observed for comfort in providing treatment to children with MIH (Figures S6 and S7), referral of children with MIH to a PD (Figures S8 and S9), or training in MIH (Figures S14 and S15). Regional analyses, including those restricted to Asia (Figures S11 and S15), consistently showed symmetrical distributions, supporting the robustness of the pooled estimates in these subgroups.

## 4. Discussion

This systematic review provides a comprehensive global overview of dental professionals’ knowledge, diagnostic confidence, clinical comfort, referral patterns and perceived need for further education related to MIH.

It should be acknowledged that the included studies used the terms knowledge and awareness inconsistently, and these constructs were frequently assessed through self-reported questionnaires rather than objective knowledge tests. The terminology adopted in this review follows that used in the primary studies.

Overall, the findings of the present meta-analysis are largely consistent with those of previous qualitative reviews, while also providing quantitative pooled estimates that refine and expand earlier conclusions.

While the overall reported data points to substantial awareness, high heterogeneity and evidence of publication bias in several domains (awareness, diagnostic confidence, caries perception), warrant cautious interpretation of the pooled estimates.

PDs consistently exhibited the highest confidence and comfort levels across all domains, from initial diagnosis to treatment provision.

Our findings highlight considerable variability across regions and professional groups, underscoring persistent gaps in the understanding and clinical management of MIH despite increasing scientific attention and a growing body of epidemiological evidence [9,12,20,21].

Our findings quantitatively corroborate the qualitative conclusions of the last systematic review [22], confirming substantial global variability in MIH knowledge, diagnostic confidence, and clinical practice. While both studies identified pediatric dentists as the most confident and knowledgeable group, our meta-analysis further demonstrated high residual heterogeneity and regional disparities, underscoring the need for standardized diagnostic criteria and structured training pathways.

### 4.1. Knowledge and Diagnostic Awareness of MIH

Overall, the level of knowledge regarding MIH diagnostic criteria and differential diagnosis among oral health professionals was moderate (72.1%). Substantial regional differences were observed, with markedly higher awareness in Oceania than in America, Europe and Asia. As this pattern is consistent with previous reports suggesting that the availability of structured national guidelines—particularly in Australia—it is possible that it may enhance clinical recognition and standardization in management [23].

Pediatric dentists consistently demonstrated higher diagnostic knowledge (94.8%) than general dental practitioners (67.3%) and other specialists (74.4%), echoing findings from earlier national surveys and cross-sectional studies [10]. This likely reflects both greater exposure to pediatric populations and more intensive postgraduate training in the developmental defects of enamel [3].

### 4.2. MIH and Caries Risk Perception

A strong body of evidence supports the increased risk of caries in MIH-affected molars [7,24,25] which is consistent with our findings, as most clinicians perceived MIH-affected teeth to be more susceptible. Nevertheless, a substantial proportion of GPD and ODS reported lower awareness of the need for specific preventive measures once MIH was identified. This gap is clinically significant since early preventive intervention is essential to minimize the progression of structural breakdown and avoid the well-documented cycle of repeated restorative failure in MIH [5,8].

### 4.3. Diagnostic and Clinical Management Confidence

Although most professionals reported some degree of confidence in diagnosing MIH, this confidence was unevenly distributed. MIH has a heterogeneous clinical presentation, ranging from subtle white opacities to extensive post-eruptive enamel breakdown, and can resemble other enamel defects. Additionally, the inconsistent application of diagnostic criteria across studies and clinical settings likely contributes to the variability in self-reported diagnostic confidence. This may partially explain the variability observed [26].

Clinicians from Oceania reported the highest diagnostic assurance, whereas those from Asian countries reported the lowest. Pediatric dentists demonstrated substantially higher diagnostic confidence than other clinicians, mirroring previous evidence that confidence correlates strongly with both training and clinical exposure [6,10].

### 4.4. Comfort Providing Care

Patients with MIH often require extensive dental treatment and generally exhibit greater apprehension in dental settings than their MIH-negative counterparts [8]. Consequently, clinicians may face behavior management complications, such as fear and anxiety attributed to hypersensitivity and pain. Additional challenges, such as reduced anesthetic efficacy and compromised adhesion to the affected enamel [4,27,28], likely contribute to the lower comfort levels reported by GDP and highlight the need for improved education and clinical training.

### 4.5. Referral Practices

Current guidelines encourage the use of all available treatment options. However, managing compromised first permanent molars can be challenging [29]. In severe cases, a multidisciplinary approach is required and referral to specialists (e.g., pediatric dentists and orthodontists) should be considered [6].

Referral behavior varied considerably. Approximately half of the dentists referred children with MIH to pediatric specialists. The heterogeneity of referral rates may be influenced by different healthcare models.

Some of the highest referral rates were observed in countries where pediatric dentistry is integrated into public health systems, such as Norway and Australia, which may be explained by the easier access to specialists. Interestingly, dental therapists/oral hygienists and postgraduate trainees exhibited the highest referral tendencies, likely reflecting their limited clinical experience and greater caution in managing complex cases.

However, given the well-documented difficulty in managing MIH, often requiring advanced behavioral management and restorative expertise, guidance on appropriate referral thresholds is warranted [3].

### 4.6. Need for Training and Implications for Practice

Most practitioners reported a need for additional education regarding MIH, with regional and professional differences suggesting uneven integration of MIH-related education in dental curricula and continuing professional development.

This aligns with international concerns about insufficient MIH coverage in undergraduate curricula and variability in postgraduate training [10,30]. Given the global prevalence of MIH and the complex clinical challenges associated with its management, this finding underscores the urgent need for standardized educational frameworks and evidence-based guidelines to support clinicians in achieving accurate diagnosis and effective treatment.

### 4.7. Strengths and Limitations

This review offers the most comprehensive global synthesis to date of MIH knowledge and clinical attitudes among dental professionals, pooling data from 29 countries. However, several limitations must be acknowledged. First, the studies included relied predominantly on self-reported questionnaires, introducing possible recall and social desirability biases. Second, the lack of standardized measurement instruments—often lacking validation—limits comparability across studies and may have substantially contributed to the heterogeneity observed. Third, the high heterogeneity observed in most meta-analyses likely reflects the combined influence of methodological differences, variability in professional experience, differences in healthcare systems, and the absence of standardized questionnaires across studies. Furthermore, the included studies were conducted over a long period (2008–2024). As awareness of MIH, educational initiatives, and clinical guidance have evolved over time, older studies may not fully reflect current levels of knowledge and clinical practice, which may also have contributed to the observed heterogeneity. Finally, the cross-sectional nature of all included studies precludes causal inferences.

### 4.8. Future Directions

Given the global burden of MIH and the substantial variation in dentists’ knowledge and confidence, future studies should prioritize the development and validation of standardized assessment tools for MIH knowledge and diagnostic proficiency. Longitudinal research is needed to evaluate the impact of targeted educational interventions on clinical behavior and patient outcomes. Additionally, research exploring structural barriers, such as access to specialists or the availability of continuing education, would provide valuable insight for oral health policymakers.

This systematic review and meta-analysis show that, despite generally adequate awareness of MIH among dental professionals, important gaps remain in diagnostic confidence, clinical management, and referral practices. These limitations are more evident among general dental practitioners and non-pediatric specialists and vary across geographic regions. The findings highlight the need for more consistent integration of MIH-related content into undergraduate education and continuing professional development, as well as greater harmonization of diagnostic and management approaches, to support more uniform and effective care for affected children.

## 5. Conclusions

This meta-analysis indicates that many children with MIH are initially managed by clinicians who report limited diagnostic confidence. While the included studies did not assess clinical performance or patient outcomes, these findings may indicate potential challenges in the timely recognition and early management of the condition.

The findings emphasize the key role of paediatric dentists in managing MIH-related challenges. Clear and standardized referral guidelines should be implemented.

The results support timely referral to paediatric dental specialists to reduce restorative failure, improve child cooperation, and optimize long-term outcomes in MIH-affected teeth.

## Figures and Tables

**Table 1:** Characteristics of included studies.

Author/Year	Country	Study Type	Sample Size (n)					Data Collection Methods	Validated Instrument	Response Rate (%)	Need for Further Training				Study Quality	Funding
											Feel the Need for Further Education and Training					
			Total	GPD	PD	ODS	Other Categories *				GPD	PD	ODS	Other Categories *		
Raj et al., 2023	India	cross-sectional study	452	92	171	189	-	Online questionnaire	Yes	90.4%	88 (95.7%)	163 (95.3%)	169 (89.4%)	NR	Low	No
Onsuren et al., 2025	Turkey	cross-sectional study	305	-	305	-	-	Online questionnaire	No	NR	NR	NR	NR	NR	Low	No
Humphreys et al., 2021	UK	cross-sectional study	76	76	-	-	-	Online questionnaire	No	NR	NR	NR	NR	NR	Moderade	NR
Hussein et al., 2024	Jordan	cross-sectional study	388	243	62	83	-	Online questionnaire	No	24.25%	287 (74.0%)	NR	NR	NR	Moderade	No
Papanikolaou et al., 2024	Holand	cross-sectional study	205	157	19	29	-	Online questionnaire Paper questionnaire	Yes	22.7%	(67.3%)	NR	NR	NR	Moderade	No
Mc Carra et al., 2023	Republic of Ireland	cross-sectional study	279	279	-	-	-	Online questionnaire	Yes	17%	NR	NR	NR	NR	Low	NR
Seremidi et al., 2022	Greek	cross-sectional study	360	185	59	116	-	Online questionnaire	Yes	94	(65%)	NR	NR	NR	Moderade	Yes
Skaare et al., 2021	Norway	cross-sectional study	100	63	-	-	37	Online questionnaire	Yes	74.6	43 (68.3%)	NR	NR	26 (70.3%)	Moderade	Yes
Wall et al., 2020	Republic of Ireland	cross-sectional study	230	230	-	-	-	Online questionnaire	No	NR	NR	NR	NR	NR	Moderade	NR
Alanzi et al., 2018	Kuwait	cross-sectional study	221	115	41	65	-	Online questionnaire	No	71.3%	39 (33.9%)	17 (41.5%)	24 (36.9%)	NR	Moderade	No
Kalkani et al., 2016	UK	cross-sectional study	68	31	37	-	-	Online questionnaire	No	71% (PD) NR (GDP)	NR	NR	NR	NR	Moderade	NR
Hussein et al., 2014	Malásia	cross-sectional study	131	97	-	-	34	Paper questionnaire	Yes	58.2%.	94 (96.9%)	NR	NR	30 (88.2%)	Moderade	NR
Ghanim et al., 2011	Iraque	cross-sectional study	146	45	-	95	-	Paper questionnaire	Yes	77.7%	30 (69.8%)	NR	79 (83%)	NR	Moderade	Yes
Bagher et al., 2025	Saudi Arabia	cross-sectional study	109	41	40	28	-	Online questionnaire	No	NR	NR	NR	NR	NR	Moderade	No
Marquillier et al., 2025	France	cross-sectional study	311	199	88	24	-	Online questionnaire	Yes	NR	NR	NR	NR	NR	Low	No
Ostermann et al., 2025	Germany	cross-sectional study	517	323	-	-	-	Online questionnaire	No	NR	400 (77.8%)	NR	NR	NR	Moderade	Yes
da Costa Rosa et al., 2024	Brasil	cross-sectional study	100	-	-	-	-	Online questionnaire	No	67.1%	99 (99%)	NR	NR	NR	Moderade	Yes
Bardellini et al., 2024	Italy	cross-sectional study	315	-	-	-	-	online questionnaire	Yes	31.5%	NR	NR	NR	NR	Moderade	No
Salerno et al., 2024	Italy	cross-sectional study	5017	-	-	-	-	online questionnaire	Yes	7.85%	NR	NR	NR	NR	Low	No
Tarhuni et al., 2023	Libya	cross-sectional study	389	389	-	-	-	Paper questionnaire	Yes	76.12%	NR	NR	NR	NR	Low	No
Gómez-Clavel et al., 2023	Mexico	cross-sectional study	391	224	67	100	-	Online questionnaire	yes	38%	NR	NR	NR	NR	Low	No
Hamza et al., 2023	Siria	cross-sectional study	1142	-	74	201	867	Online questionnaire	Yes	Students 28.9% PD 87.1% ODS 29.0%	NR	63 (85%)	97 (48%)	NR	Low	No
Costa et al., 2023	Brasil	cross-sectional study	540	61	333	146	-	Online questionnaire	No	NR	NR	NR	NR	NR	Moderade	No
Karkoutly et al., 2022	Syria	cross-sectional study	703	578	125	-	-	Online questionnaire	Yes	36.31%	NR	NR	NR	NR	Moderade	No
Delgado et al., 2022	Portugal	cross-sectional study	257	130	24	103	-	Online questionnaire	Yes	2.21%	128 (98.5%)	22 (91.7%)	91 (88.3%)	NR	Low	Yes
Negrescu et al., 2022	USA	cross-sectional study	30	4	9	17	-	NR	Yes	91%	66%	NR	NR	NR	Low	No
Liu et al., 2022	China	cross-sectional study	231	231	-	-	-	Online questionnaire	Yes	68%	90%	NR	NR	NR	Moderade	Yes
Sajadi et al.,2021	Iran	cross-sectional study	400	327	-	73	-	Paper questionnaire	Yes	NR	NR	NR	NR	NR	Moderade	Yes
Serna-Muñoz et al., 2020	Spain	cross-sectional study	214	148	66	-	-	Online questionnaire	No	18.66%	NR	NR	NR	NR	Moderade	No
Craveia et al., 2020	France	cross-sectional study	368	336	-	32	-	Online questionnaire	No	15.3%	305 (83%)	NR	NR	NR	Moderade	NR
Gamboa et al., 2018	China	cross-sectional study	255	228	27	-	-	Paper questionnaire	Yes	43.37%	201 (89%)	23 (85%)	NR	NR	Low	Yes
Tagelsir et al., 2018	USA	cross-sectional study	251	-	251	-	-	Online questionnaire	Yes	26%	NR	NR	NR	NR	Moderade	NR
Upadhyay et al., 2018	India	cross-sectional study	393	176	217	-	-	Online questionnaire	Yes	26.2	88.5%	NR	NR	NR	Moderade	NR
Gambetta-Tessini et al., 2016	Australia Chile	cross-sectional study	290	224	-	-	66	Online questionnaire Paper questionnaire	Yes	29%	90%	NR	NR	NR	Low	Yes
											NR	NR	NR	NR		
Silva et al., 2016	Saudi Arabia	cross-sectional study	357	91	48	69	149	Online questionnaire Paper questionnaire	No	60.25%	82 (90.5%)	50 (72.1%)	NR	NR	Moderade	NR
Crombie et al., 2008	Autralia New Zealand	cross-sectional study	130	59	42	13	15	Paper questionnaire	Yes	58.8%	NR	NR	NR	NR	Low	NR

* Other categories includes dental hygienists, dental therapists and undergraduate students. These students were not considered in our analysis.

**Table 2:** Pooled estimates for six items assessed for overall MIH awareness, knowledge and perception.

MIH Related Variable	N Studies	N Patients	%	95% CI	*p*-Value	I^2^	Egger Test [SE] (*p*-Value)
Diagnostic criteria	20	10,181	72.1	59.3–82.1	<0.0001	99.0	10.78 [2.39] (0.0003)
Perception of caries pattern compared to non-MIH	13	3739	84.8	76.6–90.5	<0.0001	95.2	7.49 [2.19] (0.0057)
Comfort providing treatment	12	2536	64.1	55.3–72.1	<0.0001	95.1	2.62 [4.99] (0.6104)
Referring to a PD	12	2907	52.5	34.7–69.6	<0.0001	97.5	−0.93 [5.80] (0.8765)
Confidence in the diagnosis	23	5784	67.5	58.2–75.6	<0.0001	97.5	8.52 [3.24] (0.0157)
Training	12	4522	80.7	72.3–87.2	<0.0001	95.0	3.94 [2.65] (0.1563)

MIH—Molar–Incisor Hypomineralization.

## Data Availability

No new data were created in this study. All data analyzed are derived from published studies included in this systematic review and meta-analysis.

## Supplementary Materials

The following supporting information can be downloaded at https://www.mdpi.com/article/10.3390/jcm15145591/s1, Table S1. Reasons for exclusion of studies after full-text assessment; Table S2. Table of characteristics of the included studies; Table S3. Result for subgroup analysis on the impact of risk of bias on estimates regarding confidence in diagnosing MIH; Table S4. Result for subgroup analysis on the impact of risk of bias on estimates for the perception of pattern of caries in MIH compared to non-MIH; Table S5. Result for subgroup analysis on the impact of risk of bias on comfortable providing treatment to children with HIM; Table S6. Result for subgroup analysis on the impact of risk of bias on referring children with MIH to a Pediatric Dentist; Table S7. Result for subgroup analysis on the impact of risk of bias on the confidence in diagnosing MIH; Table S8. Result for subgroup analysis on the impact of risk of bias on training regarding MIH; Table S9. Pooled estimates for the awareness for MIH diagnostic criteria for overall, each continent and specialty; Table S10. Pooled estimates for perception of pattern of caries in MIH compared to non-MIH for overall, each continent and specialty; Table S11. Pooled estimates for the comfort providing treatment to children with MIH for overall, each continent and specialty; Table S12. Pooled estimates on referring children with MIH to a Pediatric Dentist for overall, each continent and specialty; Table S13. Pooled estimates on the confidence in the diagnosis of MIH for overall, each continent and specialty; Table S14. Pooled estimates on the training in MIH for overall, each continent and specialty; Table S15. PRISMA 2020 checklist [31]; Figure S1. Funnel plot assessing publication bias for overall pooled estimates for awareness towards MIH; Figure S2. Funnel plot assessing publication bias for General Dental Practitioners pooled estimates for awareness towards MIH; Figure S3. Funnel plot assessing publication bias for Pediatric Dentists pooled estimates for awareness towards MIH; Figure S4. Funnel plot assessing publication bias for overall pooled estimates of pattern of caries in MIH compared to non-MIH; Figure S5. Funnel plot assessing publication bias for General Dental Practitioners pooled estimates for pattern of caries in MIH compared to non-MIH; Figure S6. Funnel plot assessing publication bias for overall pooled estimates on the comfort providing treatment to children with MIH; Figure S7. Funnel plot assessing publication bias for General Dental Practitioners pooled estimates on the comfort providing treatment to children with MIH; Figure S8. Funnel plot assessing publication bias for overall pooled estimates on referring children with MIH to a Pediatric Dentist; Figure S9. Funnel plot assessing publication bias for General Dental Practitioners pooled estimates on referring children with MIH to a Pediatric Dentist; Figure S10. Funnel plot assessing publication bias for overall pooled estimates on the confidence in the diagnosis of MIH; Figure S11. Funnel plot assessing publication bias for overall pooled estimates on the confidence in the diagnosis of MIH in studies from Asia; Figure S12. Funnel plot assessing publication bias for General Dental Practitioners pooled estimates on the confidence in the diagnosis of MIH; Figure S13. Funnel plot assessing publication bias for Pediatric Dentists pooled estimates on the confidence in the diagnosis of MIH; Figure S14. Funnel plot assessing publication bias for overall pooled estimates on training in MIH; Figure S15. Funnel plot assessing publication bias for overall pooled estimates on training in MIH in studies from Asia.

## Abbreviations

The following abbreviations are used in this manuscript:

MIH	Molar Incisor Hypomineralization
DDE	Developmental Defects of Enamel
CI	Confidence Interval
RoB	Risk of bias
