# Supplementary material for: Knowledge, Perception, and Clinical Experiences on Molar Incisor Hypomineralization Amongst Dental Professionals: A Systematic Review and Meta-Analysis
Source: J Clin Med. 2026 Jul 16;15(14):5591. doi: 10.3390/jcm15145591 (PMC13412580; doi:10.3390/jcm15145591)
Supplement: Supplementary file 1 [file jcm-15-05591-s001.zip › jcm-4416563-supplementary.pdf]

Knowledge, perception, and clinical experiences on molar incisor hypomineralization amongst dental professionals: a systematic review and meta-analysis

Gabriela Balixa [1], Carlota Rodrigues [1], João Botelho [1], Vanessa Machado [1], Luísa Lopes [1]

1. Egas Moniz Center for Interdisciplinary Research (CiiEM), Egas Moniz School of Health & Science, 2829-511 Almada, Portugal

Corresponding author

Gabriela Balixa

gbalixa@egasmoniz.edu.pt

Egas Moniz Center for Interdisciplinary Research (CiiEM), Egas Moniz School of Health & Science, 2829-511 Almada, Portugal

Supplementary Table S1. Reasons for exclusion of studies after full-text assessment.

Reference	Reason
Weerheijm, K. L., & Mejàre, I. (2003). Molar incisor hypomineralization: a questionnaire inventory of its occurrence in member countries of the European Academy of Paediatric Dentistry (EAPD). *International journal of paediatric dentistry*, *13*(6), 411–416. https://doi.org/10.1046/j.1365-263x.2003.00498.x	Wrong objective
Giannetti, L., Murianni, F., & Cavallini, C. (2018). MIH: A survey amongst dental practitioners in Modena and Reggio Emilia districts. *European Journal of Paediatric Dentistry*, *19*(1), 5-9.	Written in Italian. Full text not available in English.
Paes, A. F. D. (2023). Hipomineralização molar-incisivo (HMI) na Odontoclínica Central da Polícia Militar do Rio de Janeiro: prevalência, fatores relacionados e conhecimento dos cirurgiões dentistas.	Master's thesis.
Gomes, T. A. (2022). Percepção, conhecimento e experiência de cirurgiões-dentistas da Estratégia Saúde da Família do estado do Rio de Janeiro em relação ao manejo clínico da Hipomineralização Molar Incisivo.	Master's thesis.

Supplementary Table S2. Table of characteristics of the included studies.

Author / Year	Country	Study Type	Sample Size (n)					Participants	Study Objective	Data Collection Methods	Inclusion/Exclusion Criteria		Validated Instrument	Validated Instrument6	Response Rate (%)
			Total	GPD	PD	ODS	Other categories*				Inclusion	Exclusion			
Raj et al., 2023	India	cross-sectional study	452	92	171	189	-	GPD<br>PD<br>Others dental specialists	Assess and compare the knowledge, perceptions, and clinical experience about MIH	Online questionnaire	PD, GDP and Others dental specialists practicing in Northern India	Not practising dentistry or studied overseas	Yes	K. Gambetta-Tessini	90,4%
Onsuren et al., 2025	Turkey	cross-sectional study	305	-	305	-	-	PD<br>Specializing in PD<br>PhD students	Assess the knowledge, perceptions, practices, clinical experiences and attitudes of participants in pediatric dentistry specialized programs, PhD programs and among specialists regarding MIH	Online questionnaire	PD working in Turkey	NR	No	K. Gambetta-Tessini	NR
Humphreys et al., 2021	UK	cross-sectional study	76	76	-	-	-	GDP	Assess the knowledgeto to diagnose MIH when presented with multiple clinical vignettes	Online questionnaire	GDP who regularly treated children in UK	Dentists who were registered as specialists in any dental specialty were excluded from the study	No	J. Humphreys	NR
Hussein et al., 2024	Jordan	cross-sectional study	388	243	62	83	-	GDP<br>PD<br>DS	Assess the knowledge, and treatment of MIH	Online questionnaire	GPD registered in Jordan Dental Association and working in Jordan	NR	No	A. Hussein et al.	24.25%
Papanikolaou et al., 2024	Holand	cross-sectional study	205	157	19	29	-	GDP<br>PD<br>Dental Specialists	Aassess the knowledge of and attitudes towards the management of MIH amongst dentists	Online questionnaire<br>Paper questionnaire	Dentists registered in Royal Dutch Dental Association	NR	Yes	Weerheijm KL et al<br>+<br>Craveia J et al.	22,7%
Mc Carra et al., 2023	Republic of Ireland	cross-sectional study	279	279	-	-	-	GDP	Assess the knowledge, and treatment of HSPM	Online questionnaire	GPD	All Dental Specialists	Yes	K. Gambetta-Tessini	17%
Seremidi et al., 2022	Greek	cross-sectional study	360	185	59	116	-	GDP<br>PD<br>DS	Assess knowledge to diagnose and MIH and correlate findings with non-dental characteristics	Online questionnaire	Dentists members of the three largest Greek Dental Associations (Athens, Piraeus and Thessaloniki)	NR	Yes	Seremedi	94
Skaare et al., 2021	Norway	cross-sectional study	100	63	-	-	37	GDP<br>Dental Hygienists	Assess knowledge, perceptions, clinical experience and treatment options regarding MIH	Online questionnaire	GPD and Dental Hygienists working in the Public Dental Service in Oslo	NR	Yes	K. Gambetta-Tessini1	74.6
Wall et al., 2020	Republic of Ireland	cross-sectional study	230	230	-	-	-	GPD	Assess knowledge to diagnose, perceive and manage MIH	Online questionnaire	Dentists members of the Irish Dental Association Employed by the community Public Dental Service	Specialists were excluded	No	K. Gambetta-Tessini<br>entre outros	NR
Alanzi et al., 2018	Kuwait	cross-sectional study	221	115	41	65	-	GDP<br>PD<br>DS	Assess the knowledge to diagnose, prevalence, severity and clinical management oh MIH	Online questionnaire	GPD and Dental specialists members of the Kuwait and provided oral health care for children	Not practising dentistry in Kuwait	No	Crombie FA, + Ghanim A, +Bagheri R, + Hussein A, +Silva MJ	71.3%
Kalkani et al., 2016	UK	cross-sectional study	68	31	37	-	-	GPD<br>PD	Assess the knowledge, views and experience of paediatric dentistry and compare with general dental practitioners.	Online questionnaire	Specialty trainees from different paediatric dental departments in the UK<br>GPD who treated children regularly	NR	No	M. Kalkani et al.	71% (PD) NR (GDP)
Hussein et al., 2014	Malásia	cross-sectional study	131	97	-	-	34	GDP<br>Dental Nurses	Evaluate and compare the knowledge of GDP and dental nurses practicing regarding the prevalence, diagnosis, possible aetiological factors and its treatment modalities.	Paper questionnaire	GDP and Dental nurses who provide oral health care in Malaysia	Incomplete questionnaires or unanswered sections<br>Dental specialists<br>Practice outside Malaysia	Yes	Ghanim et al.	58.2%.
Ghanim et al., 2011	Iraque	cross-sectional study	146	45	-	95	-	GDP<br>Dental Specialists	Assess the perception about MIH prevalence, severity and aetiological factors.	Paper questionnaire	Teaching dental staff, including GDP and dental specialists, of Mosul Dental College.	NR	Yes	Ghanim et al.	77,7%
Bagher et al., 2025	Saudi Arabia	cross-sectional study	109	41	40	28	-	GDP<br>PD<br>Dental Specilist	Assess variations in treatment decisions made by dentists from different specialties	Online questionnaire	GPD, PD and Other Dental Specialists	Other specialties besides those mentioned	No	Sara M. Bagher	NR
Marquillier et al., 2025	France	cross-sectional study	311	199	88	24	-	Dentists in private and public practices.	Assess the knowledge, attitudes, and practices regarding MIH	Online questionnaire	Dentists members of the French Dental Practice Research Network; Dentists members of the "French pediatric dentists" social network; Dentists members of LinkedIn	NR	Yes	Bekes et al.	NR
Ostermann et al., 2025	Germany	cross-sectional study	517	323	-	-	-	GPD<br>PD<br>Other specialties (other specialties)	Assess the knowledge regarding MIH	Online questionnaire	German dentists	NR	No	Weerheijm et al.	NR
da Costa Rosa et al., 2024	Brasil	cross-sectional study	100	-	-	-	-	Dentists in private and publc practises	Evaluate and compare the perceptions, attitudes, and clinical experiences dental practitioners working in public and private health service	Online questionnaire	GPD residing in Brazil and providing clinical dental care for children and adolescents		No	Serna-Muñoz et al.	67.1%

Supplementary Table S2. Table of characteristics of the included studies (cont.).

Author / Year	Knowledge								Clinical experience												Need for further training				Study Quality	Funding
	Diagnostic criteria				Different pattern of caries on MIH				Confidence in the diagnosis				fort providing treatment to children with				efer children with HIM to a pediatric denti				l the need for further education and train					
	GPD	PD	ODS	ther categories	GPD	PD	ODS	ther categories	GPD	PD	ODS	her categories	GPD	PD	ODS	her categories	GPD	PD	ODS	ther categories	GPD	PD	ODS	ther categories		
Raj et al., 2023	74 (80,4%)	168 (98,2%)	157 (83,1%)	NR	62 (67,4%)	136 (79,5%)	120 (63,5%)	NR	45 (49%)	139 (81,3%)	130 (68,8%)	NR	74 (80,4%)	155 (90,6%)	138 (73%)	NR	74 (80,4%)	149 (87,1%)	141 (74,6%)	NR	88 (95,7%)	163 (95,3%)	169 (89,4%)	NR	Low	No
Oaruran et al., 2025	NR	243 (79,7%)	NR	NR	NR	NR	NR	NR	NR	233 (76,4%)	NR	NR	NR	NR	NR	NR	NR	NR	NR	NR	NR	NR	NR	NR	Low	No
Humphreys et al., 2021	NR	NR	NR	NR	NR	NR	NR	NR	57 (71,93%)	NR	NR	NR	NR	NR	NR	NR	NR	NR	NR	NR	NR	NR	NR	NR	Moderate	NR
Hussein et al., 2024	94 (38.7%)	52 (83.9%)	47 (56.6%)	NR	NR	NR	NR	NR	138 (56,8%)	62 (100%)	50 (60,2%)	NR	NR	NR	NR	NR	NR	NR	NR	NR	287 (74.0%)	NR	NR	NR	Moderate	No
Papanikolaou et al., 2024	6 (4%)	9 (47,4%)	5 (16,1%)	NR	NR	NR	NR	NR	NR	NR	NR	NR	(55,1%)	NR	NR	NR	2,80%	NR	NR	NR	(67,3%)	NR	NR	NR	Moderate	No
Mc Carra et al., 2023	NR	NR	NR	NR	268 (96%)	NR	NR	NR	198 (71%)	NR	NR	NR	209 (75%)	NR	NR	NR	64 (23%)	NR	NR	NR	NR	NR	NR	NR	Low	NR
Seramidi et al., 2022	168 (91%)	59 (100%)	105 (90,5%)	NR	NR	NR	NR	NR	NR	NR	NR	NR	NR	NR	NR	NR	NR	NR	NR	NR	(65%)	NR	NR	NR	Moderate	Yes
Skaare et al., 2021	57 (90.5%)	NR	NR	30 (81.1%)	54 (85.7%)	NR	NR	27 (73%)	53 (84%)	NR	NR	33 (89%9)	43 (68.3%)	NR	NR	NR	52 (82,6%)	NR	NR	NR	43 (68.3%)	NR	NR	26 (70.3%)	Moderate	Yes
Wall et al., 2020	NR	NR	NR	NR	200 (98%)	NR	NR	NR	186 (91%)	NR	NR	NR	145 (71%)	NR	NR	NR	NR	NR	NR	NR	NR	NR	NR	NR	Moderate	NR
Alanzi et al., 2018	93 (80.9%)	41 (100%)	55 (84.6%)	NR	NR	NR	NR	NR	32 (27,8%)	39 (95,1%)	60 (92,3%)	NR	NR	NR	NR	NR	NR	NR	NR	NR	39 (33.9%)	17 (41.5%)	24 (36.9%)	NR	Moderate	No
Kalkani et al., 2016	NR	NR	NR	NR	NR	NR	NR	NR	17(55%)	34(92%	NR	NR	NR	NR	NR	NR	NR	NR	NR	NR	NR	NR	NR	NR	Moderate	NR
Hussein et al., 2014	NR	NR	NR	NR	NR	NR	NR	NR	53 (54,6%)	NR	NR	25 (73,6%)	72 (74.2%)	NR	NR	32 (94.1%)	40 (57.1%)	NR	NR	30 (88.2%)	94 (96.9 %)	NR	NR	30 (88.2 %)	Moderate	NR
Ghanim et al., 2011	NR	NR	NR	NR	NR	NR	NR	NR	22 (51,1%)	NR	84 (88%)	NR	NR	NR	NR	NR	NR	NR	NR	NR	30 (69,8%)	NR	79 (83%)	NR	Moderate	Yes
Bagher et al., 2025	NR	NR	NR	NR	NR	NR	NR	NR	NR	NR	NR	NR	NR	NR	NR	NR	NR	NR	NR	NR	NR	NR	NR	NR	Moderate	No
Marquillier et al., 2025	114 (51%)	79 (89,8%)	NR	NR	NR	NR	NR	NR	181 (81%)	85 (97%)	NR	NR	NR	NR	NR	NR	NR	NR	NR	NR	NR	NR	NR	NR	Low	No
Ostermann et al., 2025	NR	NR	NR	NR	NR	NR	NR	NR	468 (91%)	NR	NR	NR	NR	NR	NR	NR	155 (36,5%)	NR	NR	NR	400 (77,8%)	NR	NR	NR	Moderate	Yes
da Costa Rosa et al., 2024	71 (71%)	NR	NR	NR	88 (88%)	NR	NR	NR	NR	NR	NR	NR	48 (48%)	NR	NR	NR	NR	NR	NR	NR	99 (99%)	NR	NR	NR	Moderate	Yes

Supplementary Table S2. Table of characteristics of the included studies (cont.).

Author / Year	Country	Study Type	Sample Size (n)					Participants	Study Objective	Data Collection Methods	Inclusion/Exclusion Criteria		Validated Instrument	Validated Instrument6	Response Rate (%)
			Total	GPD	PD	ODS	Other categories*				Inclusion	Exclusion			
**Bardellini et al., 2024**	Italy	cross-sectional study	315	-	-	-	-	Dentists	Assess the awareness of MIH and HSPM	online questionnaire	Italian dentists registered in the local National Dental Association (ANDI Brescia); Italian dentists registered in social networks.	NR	Yes	Bardellini et al.	31.5%
**Salerno et al., 2024**	Italy	cross-sectional study	5017	-	-	-	-	Dentists	Assess knowledge of DDEs, ability to recognise the different clinical pictures, and choose the most appropriate clinical approach.	online questionnaire	Italian dentists registered in the Italian Federation of Medical Doctors and Dentists	Dentists who did not sign the consent	Yes	Cagetti MG et al	7.85%
**Tarhuni et al., 2023**	Libya	cross-sectional study	389	389	-	-	-	GPD	Assess the perception regarding the prevalence, management and aetiological factors of MIH	Paper questionnaire	Libyan dentists and specialists practising dentistry in the city of Benghazi.	Incomplete questionnaires or unanswered sections	Yes	Crombie FA, + Ghanim A, + Weerheijm KL, + Tagelsir A, + Wall A, + Gamboa GCS	76,12%
**Gómez-Clavel et al., 2023**	Mexico	cross-sectional study	391	224	67	100	-	GPD<br>PD<br>DS	Assess the knowledge, experience, and perception about detection, assessment, and treatment of MIH	Online questionnaire	Dentists with postal codes corresponding to the area of interest	NR	yes	K. Gambetta-Tessini1*, R. Mariño1, A. Ghanim1, H. Calache2 and D. J. Manton1	38%
**Hamza et al., 2023**	Siria	cross-sectional study	1142	-	74	201	867	Post-- and undergraduate students	Assess the knowledge and perception of under and postgraduate students regarding diagnosing and managing MIH	Online questionnaire	Post-- and undergraduate students in all Syrian dental schools	NR	Yes	Elhennawy K, et al.	Students 28.9%<br>PD 87.1%<br>ODS 29.0%
**Costa et al., 2023**	Brasil	cross-sectional study	540	61	333	146	-	PD<br>Master's degree<br>PhD<br>GPD	Assess the knowledge concerning clinical aspects, diagnosis and consequences about MIH/HSPM among dental practitioners with different education degrees.	Online questionnaire	Registered dentists from Brazil	Incomplete questionnaires or unanswered sections	No	Alice Pinheiro Costa et al	NR
**Karkoutly et al., 2022**	Syria	cross-sectional study	703	578	125	-	-	GDP<br>PD	Evaluate and compare the knowledge, perceptions, attitudes, and clinical experiences on MIH among DPD and PD	Online questionnaire	GPD and PD registered to the Syrian Dental Syndicate of Damascus	Incomplete questionnaires or unanswered sections	Yes	Serna-Muñoz C + Alanzi A + Kalkani M, + Hamza B,	36.31%
**Delgado et al., 2022**	Portugal	cross-sectional study	257	130	24	103	-	GDP<br>PD<br>DS	Evaluate and compare the knowledge, perception, and clinical experience towards MIH between the PD, GDP and ODS	Online questionnaire	Dentists registered in the Portuguese Dental Association<br>Dentists practicing dental medicine<br>Dentists willing to participate and complete the survey.	NR	Yes	Gambetta-Tessini et al. [	2.21%
**Negrescu et al., 2022**	USA	cross-sectional study	30	4	9	17	-	GPD<br>PD<br>Orthodontics	Assess the awareness, ability, and confidence in identifying MIH among postgraduate residents	NR	GPD, PD and Orthodontits residents at the University of Nevada at Las Vegas, School of Dental Medicine.	NR	Yes	Tagelsir et al (OU SEJA, Crombie FA, + Ghanim A, + Weerheijm KL,)	91%
**Liu et al., 2022**	China	cross-sectional study	231	231	-	-	-	Undergraduate and postgraduate students	Assess the knowledge about MIH and the attitudes towards learning more about MIH among undergraduate and postgraduate students	Online questionnaire	Undergraduate students (4º and 5º year);<br>Postgraduates students (1º, 2º, 3º year) enrolled in the School of Stomatology, Wuhan University,	NR	Yes	Liu et al.	68%
**Sajadi et al.,2021**	Iran	cross-sectional study	400	327	-	73	-	GPD<br>DS	Assess the knowledge and clinical experience of GPD dentists regarding MIH	Paper questionnaire	Dentists registered in Kerman Medical Council;<br>Dentists with at least one year of clinical experience	NR	Yes	Alanzi A, + Gambetta-Tessini K + Dantas-Neta NB	NR
**Serna-Muñoz et al., 2020**	Spain	cross-sectional study	214	148	66	-	-	GDP<br>PD	Assess the knowledge and perception about the diagnosis and management strategies of MIH of GDPs and PDs	Online questionnaire	Dentists belonging to the College of Dentists of the Region of Murcia	NR	No	Serna-Muñoz	18.66%
**Craveia et al., 2020**	France	cross-sectional study	368	336	-	32	-	GPD<br>Orthodontists	Assess the knowledge and management of MIH by dentists and orthodontists	Online questionnaire	Dentist registered in the Dental Council of Southwest France;<br>Orthodontists registered in the Society of South Western Orthodontists		No	Craveia,J. et al	15,3%
**Gamboa et al., 2018**	China	cross-sectional study	255	228	27	-	-	GPD<br>PD	Evaluate and compare the knowledge, perceptions, and clinical experiences of MIH between GPD and PD	Paper questionnaire	GDP and PD registered in the Dental Council of Hong Kong	Being part of the research team	Yes	Gambetta-Tessini et al.	43.37%
**Tagelsir et al., 2018**	USA	cross-sectional study	251	-	251	-	-	PD	Assess the knowledge, perceptions, and clinical management strategies of MIH	Online questionnaire	PD identified by the AAPD 2016 to 2017 membership directory in the 12 Midwest states	Categories of AAPD members classified as affiliate, associate, international, and student	Yes	Crombie FA, + Ghanim A, + Weerheijm KL,	26%
**Upadhyay et al., 2018**	India	cross-sectional study	393	176	217	-	-	PD<br>GDP	Evaluate the perception about prevalence, severity, and etiological factors of MIH	Online questionnaire	Indian dental surgeons throughout the country	NR	Yes	Weerheijm et al + Ghanim et al.	26,2
**Gambetta-Tessini et al., 2016**	Australia<br>Chile	cross-sectional study	290	224	-	-	66	GPD<br>Oral Health Terapists	Compare the knowledge, clinical experience and perceptions about MIH between oral health care practitioners working in public dental facilities	Online questionnaire<br>Paper questionnaire	GPD working in public dental facilities that provided oral health care for children in Australia and family health centres in Chile	NR	Yes	K. Gambetta-Tessini1*,	29%
**Silva et al., 2016**	Saudi Arabia	cross-sectional study	357	91	48	69	149	GDP<br>PD<br>Dental students<br>Dental Specialists	Assess the perception of GPD, specialist dentists and dental students regarding the prevalence, severity and aetiological factors of MIH	Online questionnaire<br>Paper questionnaire	Dentists with active membership in the Saudi Dental Society;<br>Undergraduate dental students (4º and 5ª year) at two campi in the College of Dentistry, King Saud University, Riyadh	NR	No	Weerheijm et al + Crombie et al. + Ghanim et al.	60,25%
**Crombie et al., 2008**	Autralia<br>New Zealand	cross-sectional study	130	59	42	13	15	GPD<br>PD<br>Dental students<br>Dental Specialists	Assess awareness and perceptions PD concerning MIH, and to describe current treatment strategies	Paper questionnaire	Dentists registered in the Federal Committee of the Australian and New Zealand Society of Paediatric Dentistry mailing list.	NR	Yes	Weerheijm et al.	58,8%

Supplementary Table S2. Table of characteristics of the included studies (cont.).

Author / Year	Knowledge								Clinical experience												Need for further training				Study Quality	Funding
	Diagnostic criteria				Different pattern of caries on MIH				Confidence in the diagnosis				fort providing treatment to children with				efer children with MIH to a pediatric dentis				l the need for further education and train					
	GPD	PD	ODS	ther categories	GPD	PD	ODS	ther categories	GPD	PD	ODS	her categories	GPD	PD	ODS	her categories	GPD	PD	ODS	ther categories	GPD	PD	ODS	ther categories		
Bardellini et al., 2024	NR	NR	NR	NR	NR	NR	NR	NR	NR	NR	NR	NR	NR	NR	NR	NR	NR	NR	NR	NR	NR	NR	NR	NR	Moderate	No
Salerno et al., 2024	1824 (36.36%)	NR	NR	NR	3200 (63.78%)	NR	NR	NR	NR	NR	NR	NR	NR	NR	NR	NR	NR	NR	NR	NR	NR	NR	NR	NR	Low	No
Tarhuni et al., 2023	NR	NR	NR	NR	NR	NR	NR	NR	NR	NR	NR	NR	NR	NR	NR	NR	NR	NR	NR	NR	NR	NR	NR	NR	Low	No
Gómez-Clavel et al., 2023	202 (90%)	67 (100%)	92 (92%)	NR	160 (71%)	58 (87%)	80 (80%)	NR	157 (70%)	62 (92,5%)	79 (79%)	NR	121 (54%)	59 (88%)	60 (60%)	NR	NR	NR	NR	NR	NR	NR	NR	NR	Low	No
Hamza et al., 2023	NR	72 (95%)	104 (52%)	NR	NR	NR	NR	NR	NR	55 (74%)	35 (17%)	NR	NR	NR	NR	NR	NR	NR	NR	NR	NR	63 (85%)	97 (48%)	NR	Low	No
Corte et al., 2023	NR	NR	NR	NR	48 (78.7%)	295 (88.6%)	136 (93,1%)	NR	NR	NR	NR	NR	NR	NR	NR	NR	NR	NR	NR	NR	NR	NR	NR	NR	Moderate	No
Karkoutly et al., 2022	66 (18.38%)	57 (50.89%)	NR	NR	NR	NR	NR	NR	176 (37%)	72 (57,6%)	NR	NR	NR	NR	NR	NR	191 (53.20%)	34 (30.35%)	NR	NR	NR	NR	NR	NR	Moderate	No
Delgado et al., 2022	119 (91.5%)	24 (100%)	91 (88.3%)	NR	117 (90%)	23 (95.8%)	88 (85.4%)	NR	71 (54,6%)	23 (95,9%)	55 (53,4%)	NR	41 (31,5%)	21 (87,5%)	28 (27,2%)	NR	98 (75.4%)	NR	94 (91.3%)	NR	128 (98,5%)	22 (91,7%)	91 (88,3%)	NR	Low	Yes
Negrarcu et al., 2022	NR	NR	NR	NR	NR	NR	NR	NR	1 (33,3%)	9 (100%)	7 (41,2%)	NR	NR	NR	NR	NR	NR	NR	NR	NR	66%	NR	NR	NR	Low	No
Liu et al., 2022	63 (27,3%)	NR	NR	NR	NR	NR	NR	NR	NR	NR	NR	NR	NR	NR	NR	NR	NR	NR	NR	NR	90%	NR	NR	NR	Moderate	Yes
Sajadi et al.,2021	345 (86,2%)	NR	NR	NR	317 (79,2%)	NR	NR	NR	66 (16,4%)	NR	NR	NR	NR	NR	NR	NR	195,2 (48,8%)	NR	NR	NR	NR	NR	NR	NR	Moderate	Yes
Serna-Muñoz et al., 2020	NR	NR	NR	NR	NR	NR	NR	NR	NR	NR	NR	NR	NR	NR	NR	NR	NR	NR	NR	NR	NR	NR	NR	NR	Moderate	No
Gravois et al., 2020	NR	NR	NR	NR	NR	NR	NR	NR	NR	NR	NR	NR	NR	NR	NR	NR	NR	NR	NR	NR	305 (83%)	NR	NR	NR	Moderate	NR
Gamboa et al., 2018	166 (72.8%)	26 (96.3%)	NR	NR	145 (65.9%)	22 (81.5%)	NR	NR	127 (55,7%)	25 (92,6%)	NR	NR	96 (42,5%)	23 (77,8%)	NR	NR	132 (57.9%)	14 (51.9%)	NR	NR	201 (89%)	23 (85%)	NR	NR	Low	Yes
Tagelsir et al., 2018	NR	100 (37%)	NR	NR	NR	NR	NR	NR	NR	249 (99%)	NR	NR	NR	NR	NR	NR	NR	NR	NR	NR	NR	NR	NR	NR	Moderate	NR
Upadhyay et al., 2018	NR	NR	NR	NR	NR	NR	NR	NR	NR	NR	NR	NR	NR	NR	NR	NR	NR	NR	NR	NR	88,5%	NR	NR	NR	Moderate	NR
mbetta-Tessini et al., 20	40 (87.0) AUSTRALIA	NR	NR	61 (92.4)	42 (89.4)	NR	NR	58 (87.9)	104 (92.9)	NR	NR	NR	93 (83.0)	NR	NR	NR	37 (78.7)	NR	NR	45 (69.2)	90%	NR	NR	NR	Low	Yes
	97 (82.9) CHILE	NR	NR	NR	107 (91.5)	NR	NR	NR	78 (83.0)	NR	NR	NR	72 (62.1)	NR	NR	NR	47 (40.5)	NR	NR	NR	NR	NR	NR	NR		
Silva et al., 2016	54 (59%)	41 (85%)	44 (64%)	NR	nR	NR	NR	NR	44 (68,7%)	39 (97,5%)	34 (49%)	NR	NR	NR	NR	NR	NR	NR	NR	NR	82 (90,5%)	50 (72,1%)	NR	NR	Moderate	NR
Crombie et al., 2008	NR	NR	NR	NR	nR	NR	NR	NR	NR	NR	NR	NR	NR	NR	NR	NR	NR	NR	NR	NR	NR	NR	NR	NR	Low	NR

Supplementary Table S3. Result for subgroup analysis on the impact of risk of bias on estimates regarding confidence in diagnosing MIH

	N studies	N	%	95% CI	p-value	I2
Risk of bias						
Low	10	7,494	79.4	68.3-87.4	<0.0001	99.2
Moderate	10	2,687	63.1	40.7-81.1	<0.0001	98.7

Test for subgroup differences (random effects model): Q = 2.15; degrees of freedom = 1; p-value = 0.1423

Supplementary Table S4. Result for subgroup analysis on the impact of risk of bias on estimates for the perception of pattern of caries in MIH compared to non-MIH

	N studies	N	%	95% CI	p-value	I2
Risk of bias						
Low	8	2,359	83.2	73.0-90.1	<0.0001	94.9
Moderate	5	1,380	87.1	71.5-94.8	<0.0001	95.3

Test for subgroup differences (random effects model): Q = 0.27; degrees of freedom = 1; p-value = 0.6058

Supplementary Table S5. Result for subgroup analysis on the impact of risk of bias on comfortable providing treatment to children with HIM

	N studies	N	%	95% CI	p-value	I2
Risk of bias						
Low	11	2,765	71.6	60.7-80.5	<0.0001	96.1
Moderate	12	3,019	63.4	48.9-75.9	<0.0001	97.8

Test for subgroup differences (random effects model): Q = 0.91; degrees of freedom = 1; p-value = 0.3410

Supplementary Table S6. Result for subgroup analysis on the impact of risk of bias on referring children with MIH to a Pediatric Dentist

	N studies	N	%	95% CI	p-value	I2

Risk of bias						
Low	6	1,416	64.9	41.3-77.7	<0.0001	98.0
Moderate	6	1,491	43.6	19.4-71.3	<0.0001	96.7

Test for subgroup differences (random effects model): Q = 0.06; degrees of freedom = 1; p-value = 0.8030

Supplementary Table S7. Result for subgroup analysis on the impact of risk of bias on the confidence in diagnosing MIH

	N studies	N	%	95% CI	p-value	I2
Risk of bias						
Low	11	2,765	71.6	60.7-80.5	<0.0001	96.1
Moderate	12	3,019	63.4	48.9-75.9	<0.0001	97.8

Test for subgroup differences (random effects model): Q = 0.91; degrees of freedom = 1; p-value = 0.3410

Supplementary Table S8. Result for subgroup analysis on the impact of risk of bias on training regarding MIH

	N studies	N	%	95% CI	p-value	I^2
Risk of bias						
Low	5	1,354	78.9	63.4-89.0	<0.0001	97.0
Moderate	13	3,168	81.7	70.9-89.2	<0.0001	94.2

Test for subgroup differences (random effects model): Q = 0.13; degrees of freedom = 1; p-value = 0.7180

Supplementary Table S9. Pooled estimates for the awareness for MIH diagnostic criteria for overall, each continent and specialty.

	N studies	N patients	%	95% CI	p-value	I^2	Egger Test [SE] (p-value)
Overall	20	10,181	72.1	59.3-82.1	<0.0001	99.0	10.78 [2.39] (0.0003)
Asia	9	2,910	66.0	48.7-80.0	<0.0001	98.7	-

Europe	7	6,551	69.4	41.4-88.2	<0.0001	99.2	-
America	3	608	84.3	71.6-91.9	<0.0001	93.5	-
Oceania	1	112	90.2	83.1-94.5	-	-	-
Specialty							
GDP	18	8,024	67.3	50.7-80.5	<0.0001	98.4	6.77 [2.05]] (0.0044)
PD	13	1,099	94.8	84.4-98.4	<0.0001	87.6	2.98 [1.15] (0.0254)
ODS	9	955	74.4	56.4-86.7	<0.0001	94.2	-

GDP – General Dental Practitioner; PD – Pediatric Dentist; ODS – Other Dental Specialists; SE – Standard Error; MIH – Molar-Incisor Hypomineralization

Supplementary Table S10. Pooled estimates for perception of pattern of caries in MIH compared to non-MIH for overall, each continent and specialty.

	N studies	N patients	%	95% CI	p-value	I^2	Egger Test [SE] (p-value)
**Overall**	13	3,739	84.8	76.6-90.5	<0.0001	95.2	7.49 [2.19] (0.0057)
Asia	3	1,099	72.8	66.7-78.2	<0.0001	84.6	-
Europe	5	1,378	88.1	68.4-96.2	<0.0001	97.3	-
America	4	1,149	86.5	79.7-91.2	<0.0001	90.8	-
Oceania	1	112	88.5	81.2-93.2	-	-	-
**Specialty**							
GDP	13	2440	85.2	77.3-90.7	<0.0001	93.1	6.03 [1.35] (0.0010)
PD	5	622	85.6	80.9-89.7	<0.0001	58.1	-
ODS	4	538	82.7	69.4-90.9	<0.0001	92.7	-

GDP – General Dental Practitioner; PD – Pediatric Dentist; ODS – Other Dental Specialists; SE – Standard Error; MIH – Molar-Incisor Hypomineralization

Supplementary Table S11. Pooled estimates for the comfort providing treatment to children with MIH for overall, each continent and specialty.

	N studies	N patients	%	95% CI	p-value	I^2	Egger Test [SE] (p-value)
Overall	12	2,536	64.1	55.3-72.1	<0.0001	95.1	2.62 [4.99] (0.6104)
Asia	3	805	54.4	41.9-66.2	<0.0001	97.7	-
Europe	5	1,012	74.3	61.8-83.7	<0.0001	95.9	-
America	3	607	76.5	72.6-79.9	<0.0001	68.1	-
Oceania	1	112	83.0	74.9-88.9	-	-	-
Specialty							
GDP	11	1,647	63.4	53.2-72.6	<0.0001	93.6	5.01 [4.85] (0.3281)
PD	4	292	88.4	84.1-91.6	<0.0001	34.6	-
ODS	3	392	53.7	31.1-74.9	<0.0001	96.1	-

GDP – General Dental Practitioner; PD – Pediatric Dentist; ODS – Other Dental Specialists; SE – Standard Error; MIH – Molar-Incisor Hypomineralization

Supplementary Table S12. Pooled estimates on referring children with MIH to a Pediatric Dentist for overall, each continent and specialty.

	N studies	N patients	%	95% CI	p-value	I^2	Egger Test [SE] (p-value)
Overall	12	2,907	52.5	34.7-69.6	<0.0001	97.5	-0.93 [5.80] (0.8765)
Asia	6	1,855	57.2	44.1-69.0	<0.0001	96.7	-
Europe	4	824	42.0	9.1-84.0	<0.0001	98.9	-
America	1	116	73.2	64.3-80.6	-	-	-

Oceania	1	112	83.0	74.9-88.9	-	-	-
Specialty							
GDP	11	2,001	58.5	45.8-70.1	<0.0001	95.2	5.66 [3.73] (0.1627)
PD	3	310	59.7	28.1-84.9	<0.0001	97.5	-
ODS	2	292	84.1	68.4-92.8	<0.0001	90.7	-

GDP – General Dental Practitioner; PD – Pediatric Dentist; ODS – Other Dental Specialists; SE – Standard Error; MIH – Molar-Incisor Hypomineralization

Supplementary Table S13. Pooled estimates on the confidence in the diagnosis of MIH for overall, each continent and specialty

	N studies	N patients	%	95% CI	p-value	I^2	Egger Test [SE] (p-value)
Overall	23	5,784	67.5	58.2-75.6	<0.0001	97.5	8.52 [3.24] (0.0157)
Asia	10	2,761	54.4	41.9-66.2	<0.0001	97.5	4.71 [8.42] (0.5914)
Europe	9	1,812	74.3	61.8-83.7	<0.0001	97.8	-
America	3	514	76.5	72.6-79.9	<0.0001	71.5	-
Oceania	1	112	92.9	86.4-96.4	-	-	-
Specialty							
GDP	20	3,202	63.3	52.1-73.2	<0.0001	96.6	1.86 [2.79] (0.0791)
PD	13	1,070	92.1	84.3-96.2	<0.0001	83.0	3.96 [0.75] (0.0022)
ODS	9	927	64.0	45.0-79.4	<0.0001	95.7	-

GDP – General Dental Practitioner; PD – Pediatric Dentist; ODS – Other Dental Specialists; SE – Standard Error; MIH – Molar-Incisor Hypomineralization

Supplementary Table S14. Pooled estimates on the need for further MIH-related training for overall, each continent and specialty

	N studies	N patients	%	95% CI	p-value	I^2	Egger Test [SE] (p-value)
Overall	12	4,522	80.7	72.3-87.2	<0.0001	95.0	3.94 [2.65] (0.1563)
Asia	10	2,587	83.2	72.2-90.4	<0.0001	96.3	1.24 [4.77] (0.2488)
Europe	5	1,529	74.4	68.4-79.5	<0.0001	85.3	-
America	3	406	58.2	51.9-64.6	<0.0001	89.1	-
Specialty							
GDP	9	957	90.5	76.0-96.6	<0.0001	95.4	-
PD	6	406	83.0	66.3-92.4	<0.0001	91.0	-
ODS	5	740	64.7	42.0-82.2	<0.0001	96.3	-

GDP – General Dental Practitioner; PD – Pediatric Dentist; ODS – Other Dental Specialists; SE – Standard Error; MIH – Molar-Incisor Hypomineralization

Supplementary Table S15. PRISMA 2020 checklist

PRISMA 2020 Checklist

Section and Topic	Item #	Checklist item	Location where item is reported
**TITLE**			
Title	1	Identify the report as a systematic review.	1
**ABSTRACT**			
Abstract	2	See the PRISMA 2020 for Abstracts checklist.	1-2
**INTRODUCTION**			
Rationale	3	Describe the rationale for the review in the context of existing knowledge.	2-3
Objectives	4	Provide an explicit statement of the objective(s) or question(s) the review addresses.	3
**METHODS**			
Eligibility criteria	5	Specify the inclusion and exclusion criteria for the review and how studies were grouped for the syntheses.	3-4
Information sources	6	Specify all databases, registers, websites, organisations, reference lists and other sources searched or consulted to identify studies. Specify the date when each source was last searched or consulted.	4
Search strategy	7	Present the full search strategies for all databases, registers and websites, including any filters and limits used.	4
Selection process	8	Specify the methods used to decide whether a study met the inclusion criteria of the review, including how many reviewers screened each record and each report retrieved, whether they worked independently, and if applicable, details of automation tools used in the process.	4-5
Data collection process	9	Specify the methods used to collect data from reports, including how many reviewers collected data from each report, whether they worked independently, any processes for obtaining or confirming data from study investigators, and if applicable, details of automation tools used in the process.	4-5
Data items	10a	List and define all outcomes for which data were sought. Specify whether all results that were compatible with each outcome domain in each study were sought (e.g. for all measures, time points, analyses), and if not, the methods used to decide which results to collect.	4-5
	10b	List and define all other variables for which data were sought (e.g. participant and intervention characteristics, funding sources). Describe any assumptions made about any missing or unclear information.	4-5
Study risk of bias assessment	11	Specify the methods used to assess risk of bias in the included studies, including details of the tool(s) used, how many reviewers assessed each study and whether they worked independently, and if applicable, details of automation tools used in the process.	5
Effect measures	12	Specify for each outcome the effect measure(s) (e.g. risk ratio, mean difference) used in the synthesis or presentation of results.	5
Synthesis methods	13a	Describe the processes used to decide which studies were eligible for each synthesis (e.g. tabulating the study intervention characteristics and comparing against the planned groups for each synthesis (item #5)).	5
	13b	Describe any methods required to prepare the data for presentation or synthesis, such as handling of missing summary statistics, or data conversions.	5
	13c	Describe any methods used to tabulate or visually display results of individual studies and syntheses.	5
	13d	Describe any methods used to synthesize results and provide a rationale for the choice(s). If meta-analysis was performed, describe the model(s), method(s) to identify the presence and extent of statistical heterogeneity, and software package(s) used.	5
	13e	Describe any methods used to explore possible causes of heterogeneity among study results (e.g. subgroup analysis, meta-regression).	5
	13f	Describe any sensitivity analyses conducted to assess robustness of the synthesized results.	5
Reporting bias assessment	14	Describe any methods used to assess risk of bias due to missing results in a synthesis (arising from reporting biases).	4-5
Certainty assessment	15	Describe any methods used to assess certainty (or confidence) in the body of evidence for an outcome.	5

PRISMA 2020 Checklist

Section and Topic	Item #	Checklist item	Location where item is reported
**RESULTS**			
Study selection	16a	Describe the results of the search and selection process, from the number of records identified in the search to the number of studies included in the review, ideally using a flow diagram.	6
	16b	Cite studies that might appear to meet the inclusion criteria, but which were excluded, and explain why they were excluded.	7
Study characteristics	17	Cite each included study and present its characteristics.	6-8
Risk of bias in studies	18	Present assessments of risk of bias for each included study.	8
Results of individual studies	19	For all outcomes, present, for each study: (a) summary statistics for each group (where appropriate) and (b) an effect estimate and its precision (e.g. confidence/credible interval), ideally using structured tables or plots.	9-11
Results of syntheses	20a	For each synthesis, briefly summarise the characteristics and risk of bias among contributing studies.	9-11
	20b	Present results of all statistical syntheses conducted. If meta-analysis was done, present for each the summary estimate and its precision (e.g. confidence/credible interval) and measures of statistical heterogeneity. If comparing groups, describe the direction of the effect.	9-11
	20c	Present results of all investigations of possible causes of heterogeneity among study results.	9-11
	20d	Present results of all sensitivity analyses conducted to assess the robustness of the synthesized results.	9-11
Reporting biases	21	Present assessments of risk of bias due to missing results (arising from reporting biases) for each synthesis assessed.	8
Certainty of evidence	22	Present assessments of certainty (or confidence) in the body of evidence for each outcome assessed.	9-11
**DISCUSSION**			
Discussion	23a	Provide a general interpretation of the results in the context of other evidence.	11-14
	23b	Discuss any limitations of the evidence included in the review.	11-14
	23c	Discuss any limitations of the review processes used.	11-14
	23d	Discuss implications of the results for practice, policy, and future research.	13-14
**OTHER INFORMATION**			
Registration and protocol	24a	Provide registration information for the review, including register name and registration number, or state that the review was not registered.	3
	24b	Indicate where the review protocol can be accessed, or state that a protocol was not prepared.	3
	24c	Describe and explain any amendments to information provided at registration or in the protocol.	3
Support	25	Describe sources of financial or non-financial support for the review, and the role of the funders or sponsors in the review.	15
Competing interests	26	Declare any competing interests of review authors.	15
Availability of data, code and other materials	27	Report which of the following are publicly available and where they can be found: template data collection forms; data extracted from included studies; data used for all analyses; analytic code; any other materials used in the review.	-

From: Page MJ, McKenzie JE, Bossuyt PM, Boutron I, Hoffmann TC, Mulrow CD, et al. The PRISMA 2020 statement: an updated guideline for reporting systematic reviews. BMJ 2021;372:n71. doi: 10.1136/bmj.n71. This work is licensed under CC BY 4.0. To view a copy of this license, visit https://creativecommons.org/licenses/by/4.0/

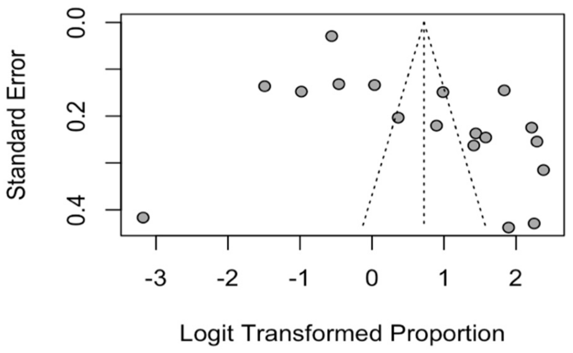

Supplementary Figure S1. Funnel plot assessing publication bias for overall pooled estimates for awareness towards MIH. Visual asymmetry suggests potential small-study effects or publication bias, which was confirmed by Egger's regression test (t = 3.31, df = 16, p = 0.0044).

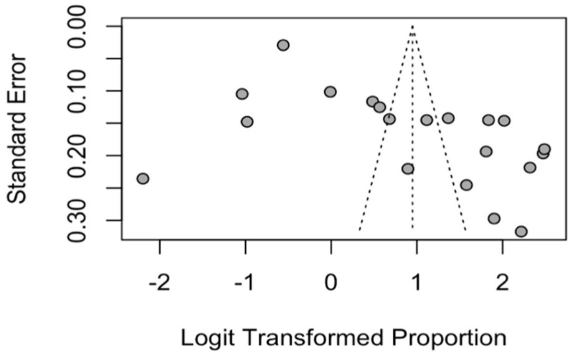

Supplementary Figure S2. Funnel plot assessing publication bias for General Dental Practitioners pooled estimates for awareness towards MIH. Visual asymmetry suggests potential small-study effects or publication bias, which was confirmed by Egger's regression test (t = 2.58, df = 11, p = 0.0254).

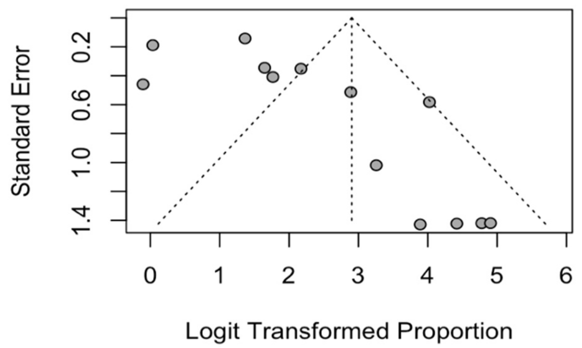

Supplementary Figure S3. Funnel plot assessing publication bias for Pediatric Dentists pooled estimates for awareness towards MIH. Visual asymmetry suggests potential small-study effects or publication bias, which was confirmed by Egger's regression test (t = 4.50, df = 18, p = 0.0003).

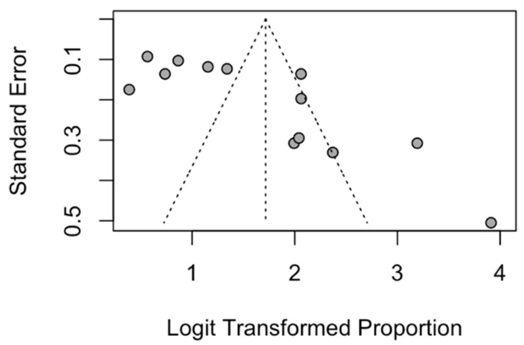

Supplementary Figure S4. Funnel plot assessing publication bias for overall pooled estimates of pattern of caries in MIH compared to non-MIH. Visual asymmetry suggests potential small-study effects or publication bias, which was confirmed by Egger's regression test (t = 4.45, df = 11, p = 0.0010).

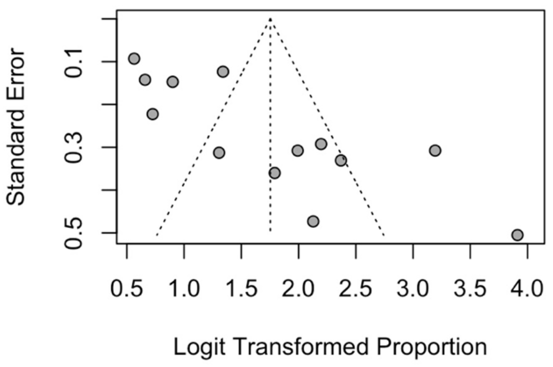

Supplementary Figure S5. Funnel plot assessing publication bias for General Dental Practitioners pooled estimates for pattern of caries in MIH compared to non-MIH. Visual asymmetry suggests potential small-study effects or publication bias, which was confirmed by Egger's regression test (t = 4.45, df = 11, p = 0.0010).

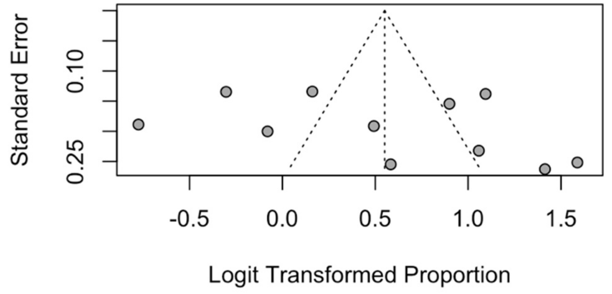

Supplementary Figure S6. Funnel plot assessing publication bias for overall pooled estimates on the comfort providing treatment to children with MIH. Visual symmetry suggests no potential small-study effects or publication bias, which was confirmed by Egger's regression test (t = 2.62, df = 10, p = 0.6104).

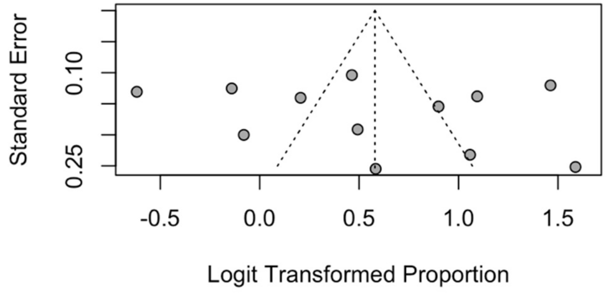

Supplementary Figure S7. Funnel plot assessing publication bias for General Dental Practitioners pooled estimates on the comfort providing treatment to children with MIH. Visual symmetry suggests no potential small-study effects or publication bias, which was confirmed by Egger's regression test (t = 1.03, df = 9, p = 0.3281).

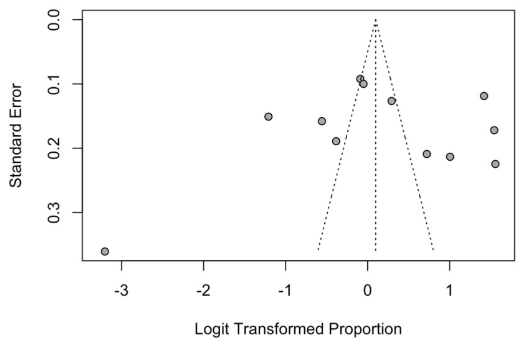

Supplementary Figure S8. Funnel plot assessing publication bias for overall pooled estimates on referring children with MIH to a Pediatric Dentist. Visual symmetry suggests no potential small-study effects or publication bias, which was confirmed by Egger's regression test (t = -0.93, df = 10, p = 0.8765).

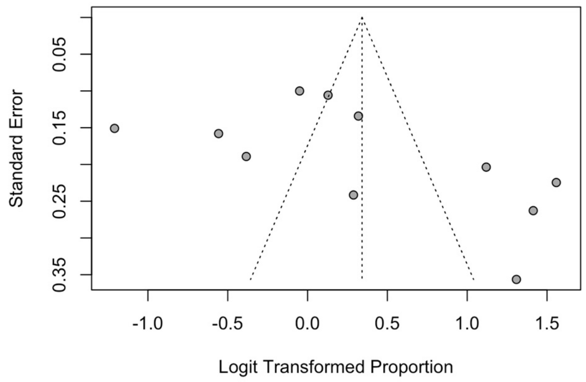

Supplementary Figure S9. Funnel plot assessing publication bias for General Dental Practitioners pooled estimates on referring children with MIH to a Pediatric Dentist. Visual symmetry suggests no potential small-study effects or publication bias, which was confirmed by Egger's regression test (t = 1.52, df = 9, p = 0.1627).

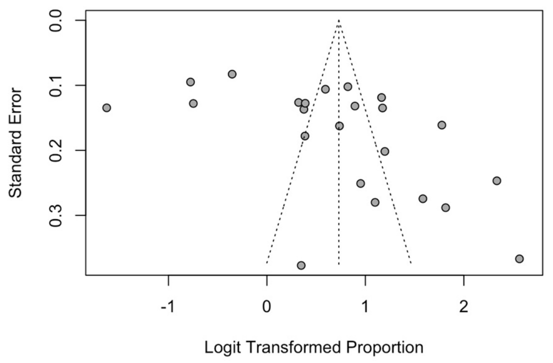

Supplementary Figure S10. Funnel plot assessing publication bias for overall pooled estimates on the confidence in the diagnosis of MIH. Visual asymmetry suggests potential small-study effects or publication bias, which was confirmed by Egger's regression test (t = 2.63, df = 21, p = 0.0157).

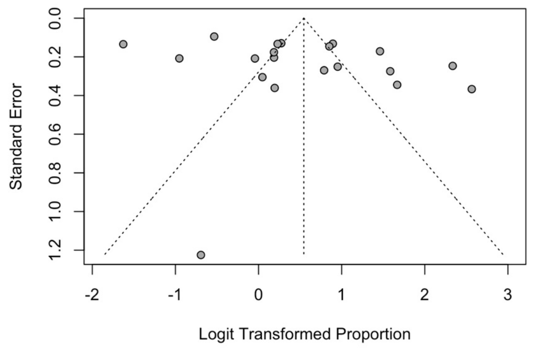

Supplementary Figure S11. Funnel plot assessing publication bias for overall pooled estimates on the confidence in the diagnosis of MIH in studies from Asia. Visual symmetry suggests no potential small-study effects or publication bias, which was confirmed by Egger's regression test (t = 0.56, df = 8, p = 0.5914).

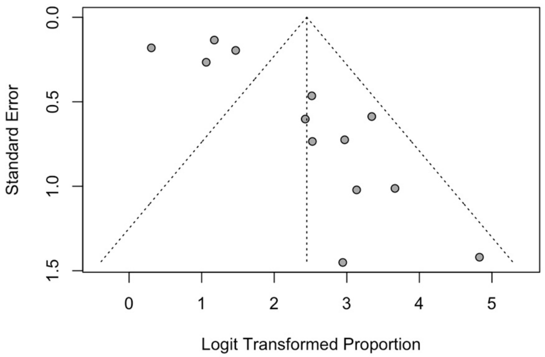

Supplementary Figure S12. Funnel plot assessing publication bias for General Dental Practitioners pooled estimates on the confidence in the diagnosis of MIH. Visual symmetry suggests no potential small-study effects or publication bias, which was confirmed by Egger's regression test (t = 1.86, df = 18, p = 0.0791).

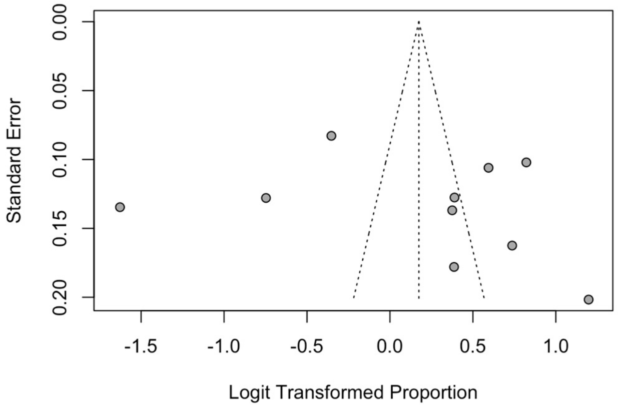

Supplementary Figure S13. Funnel plot assessing publication bias for Pediatric Dentists pooled estimates on the confidence in the diagnosis of MIH. Visual asymmetry suggests potential small-study effects or publication bias, which was confirmed by Egger's regression test (t = 3.96, df = 11, p = 0.0022).

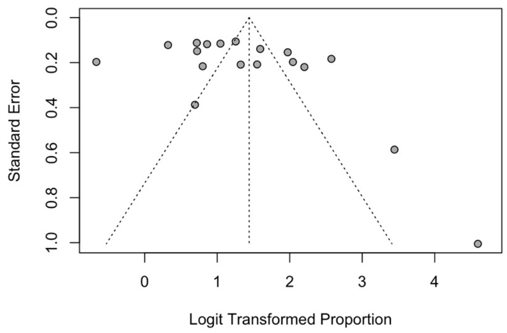

Supplementary Figure S14. Funnel plot assessing publication bias for overall pooled estimates on training in MIH. Visual symmetry suggests no potential small-study effects or publication bias, which was confirmed by Egger's regression test (t = 1.49, df = 16, p = 0.1563).

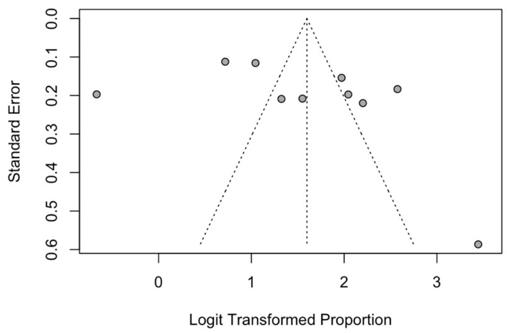

Supplementary Figure S15. Funnel plot assessing publication bias for overall pooled estimates on training in MIH in studies from Asia. Visual symmetry suggests no potential small-study effects or publication bias, which was confirmed by Egger's regression test (t = 1.24, df = 8, p = 0.2488).
